# Serological evaluation of patients with coronavirus disease-2019 in Daegu, South Korea

**DOI:** 10.1371/journal.pone.0262820

**Published:** 2022-01-20

**Authors:** Sunggyun Park, Soon Hee Chang, Jae Hee Lee, Jong Ho Lee, Ji Yeon Ham, Yu Kyung Kim, Sang-Gyung Kim, Nam Hee Ryoo

**Affiliations:** 1 Department of Laboratory Medicine, Keimyung University School of Medicine, Daegu, Korea; 2 Department of Clinical Pathology, School of Medicine, Kyungpook National University, Daegu, Korea; 3 Department of Laboratory Medicine, Keimyung University Daegu-Dongsan Hospital, Daegu, Korea; 4 Department of Laboratory Medicine, Yeungnam University College of Medicine, Daegu, Korea; 5 Department of Laboratory Medicine, Daegu Catholic University Hospital, Daegu, Korea; "INSERM", FRANCE

## Abstract

**Background:**

Early and accurate detection of severe acute respiratory syndrome coronavirus 2 (SARS-CoV-2) is critical to prevent spread of the infection. Understanding of the antibody response to SARS-CoV-2 in patients with coronavirus disease 2019 (COVID-19) is insufficient, particularly in relation to those whose responses persist for more than 1 month after the onset of symptoms. We conducted a SARS-CoV-2 antibody test to identify factors affecting the serological response and to evaluate its diagnostic utility in patients with COVID-19.

**Methods and finding:**

We collected 1,048 residual serum samples from 396 patients with COVID-19 confirmed by real-time reverse transcription polymerase chain reaction (RT-PCR) for SARS-CoV-2. The samples had been used for routine admission tests in six healthcare institutions in Daegu. Antibody to SARS-CoV-2 was analyzed and the cutoff index (COI) was calculated for quantitative analysis. The patients’ information was reviewed to evaluate the relationship between antibody positivity and clinical characteristics. The anti-SARS-CoV-2 antibody positivity rate was 85% and the average COI was 24·3. The positivity rate and COI increased with time elapsed since symptom onset. Anti-SARS-CoV-2 antibody persisted for at least 13 weeks after symptom onset at a high COI. There was a significant difference in anti-SARS-CoV-2 antibody positivity rate between patients with and without symptoms, but not according to sex or disease course. The descending COI pattern at weeks 1 to 5 after symptom onset was significantly more frequent in patients who died than in those who recovered.

**Conclusions:**

Anti-SARS-CoV-2 antibody persisted for at least 13 weeks at a high COI in patients with COVID-19. A decreasing COI pattern up to fifth week may be associated with a poor prognosis of COVID-19. As new treatments and vaccines are introduced, it is important to monitor continuously the usefulness of anti-SARS-CoV-2 antibody assays.

## Introduction

Severe acute respiratory syndrome coronavirus 2 (SARS-CoV-2) has spread rapidly worldwide since the World Health Organization (WHO) declared a pandemic on March 11, 2020 [1]. The first case of coronavirus disease 2019 (COVID-19) in South Korea was detected on January 20 and was followed by a large outbreak originating in a church in Daegu City. Early and accurate detection of SARS-CoV-2 became critical to allow quarantine of exposed persons to prevent further spread. Failure to diagnose COVID-19 promptly and accurately may delay proper treatment and increase the risk of disease transmission. Furthermore, false-positive results lead to unnecessary additional tests, treatments, and isolation of patients.

For accurate diagnosis of COVID-19, real-time reverse transcriptase polymerase chain reaction (real-time RT-PCR) and serologic assays based on antigen-antibody reactions have been introduced. Real-time RT-PCR for SARS-CoV-2 in clinical samples (*e*.*g*., nasopharyngeal swab, oropharyngeal swab, or sputum) is the gold standard diagnostic method for COVID-19 [2]. SARS-CoV-2 antibody testing is recommended as an adjunct to real-time RT-PCR assay [3]. However, real-time RT-PCR assays are hampered by an increasing rate of false negative or inconclusive results over time since symptom onset [4].

The need for a serological assay for COVID-19 has been highlighted by the limitations of real-time RT-PCR. Antibody tests for COVID-19 showed precise results due to less effect of sample collection and antigenic mutations than RT-PCR or antigen tests, and were significant in diagnosis as they could reduce the false negative or indeterminate results due to a decrease in the amount of virus in the nasopharynx after 1 week from symptom onset [5–8]. In addition, antibody testing can provide additional information about past infections, and has the advantage of being easier to set up compared to RT-PCR and easy to access in the early stages of a pandemic [9]. However, there is insufficient understanding of antibody responses to SARS-CoV-2 in patients with COVID-19, particularly in relation to those whose responses have persisted for more than 1 month after onset of symptoms. We conducted SARS-CoV-2 antibody testing on 1048 samples from 396 patients with COVID-19 in six healthcare institutions in Daegu to identify associated factors and confirm the diagnostic utility of serological testing.

## Materials and methods

### Patients and samples

We collected 1,048 residual serum samples used for routine admission tests in six healthcare institutions in Daegu: Yeungnam University Medical Center, Daegu Catholic University Medical Center, Kyungpook National University Hospital, Kyungpook National University Chilgok Hospital, Daegu Fatima hospital, and Keimyung University Dongsan Medical Center. The serum samples were from 396 patients with COVID-19 confirmed by real-time RT-PCR for SARS-CoV-2. All the serum samples were collected from all COVID-19 confirmed and admitted patients with sufficient volume of serum remained after the routine laboratory tests in each healthcare institution. An average of 2.7 samples per patient was included (maximum number of samples per single patient was 57). The clinical characteristics of the patients were reviewed using the electronic medical records. If each patient has symptoms of various respiratory infections, such as fever, malaise, cough, sputum, dyspnea and pneumonia, as well as non-specific symptoms such as sore throat, headache, hemoptysis, nausea, and diarrhea, that case was classified as the symptomatic group, and the case without acute symptoms was classified as the asymptomatic group. Time since symptom onset was based on the date on which symptoms manifested or the date of real-time RT-PCR confirmation for asymptomatic patients. The cycle threshold (Ct) value of *RdRP* was based on the first positive real-time RT-PCR result.

### Measurement of the SARS-CoV-2 antibody level

Antibody against SARS-CoV-2 was measured by Elecsys Anti-SARS-CoV-2 Electrochemiluminescence Assay (Roche Diagnostics, Rotkreuz, Switzerland) using a fully automated Cobas e801 Analyzer (Roche Diagnostics) according to the manufacturer’s instructions. The assay uses a recombinant protein representing the nucleocapsid (N) antigen in a double-antigen sandwich assay format and detects total antibodies, including IgG. We decided to identify antibodies against N antigen because there was no information about the mutation on *Spike* gene of each infected SARS-CoV-2. Results are provided as numeric cutoff index (COI) values and are finally reported as positive (COI ≥ 1) or negative (COI < 1).

### Statistical analysis

The chi-squared for trend-in-proportion test was used to compare qualitative results among more than two classes, such as number of weeks elapsed after symptom onset. Pearson’s chi-squared test with Yates’ continuity correction and Fisher’s exact test were used to compare qualitative parameters between two classes, such as sex, presence of symptoms, and disease course. To compare quantitative parameters among more than two classes, the Kruskal–Wallis rank-sum test was used with the Games–Howell nonparametric test for *post hoc* analysis. For comparisons of two classes, the Wilcoxon rank-sum test with continuity correction was used.

We performed logistic regression to compare the qualitative results (positive or negative) according to time since symptom onset and Spearman’s rank correlation to compare COI values. The slope of the linear regression equation was used to categorize ascending and descending patterns of consecutive samples. All statistical analyses were conducted using R version 4·0·2.

### Ethics statement

The study protocol was reviewed and approved by the Institutional Review Board of Medicity Daegu Joint (approval no. DGIRB 2020-05-009-003). We anonymized all of the medical records and blood samples from the patients before the assessment, and the IRB waived the requirement for informed consent because we used samples remained after the routine laboratory tests.

## Results

### Patient distribution and sample characteristics

The average age of the patients was 62 years, and the male to female ratio was 4·5. Of the patients, 79·3% and 11·3% were symptomatic and deceased, respectively. The average number of days since symptom onset was 30. The total anti-SARS-CoV-2 antibody positivity rate was 85% and the average COI was 24·3. The results for each institution are listed in Table 1.

**Table 1:** Characteristics of the patients and samples.

Institution	Number of patients	Mean age	Male to female ratio	Patients with Symptom (%)	Deceased patients (%)	Number of samples	Mean days* (min-max)	Number of positive results (%)	Mean COI (min-max)
1	30	65.8	56.67	15 (50.0)	3 (10.0)	107	40.1 (7–79)	105 (98.1)	32.6 (0.092–135)
2	60	66.6	43.33	60 (100.0)	12 (20.0)	100	25.9 (3–76)	88 (88.0)	30.3 (0.084–114)
3	60	68.7	56.67	59 (98.3)	7 (11.7)	139	28.9 (0–67)	129 (92.8)	25.7 (0.082–95.9)
4	27	65.4	48.15	22 (81.5)	8 (30.8)	214	31.4 (1–62)	202 (94.4)	20.9 (0.085–102.0)
5	165	54.2	63.03	104 (63.0)	5 (3.9)	198	27.6 (1–90)	126 (63.6)	25.0 (0.081–144)
6	54	66.2	42.59	54 (100.0)	5 (10.6)	290	28.9 (1–81)	241 (83.1)	20.7 (0.084–112)
Total	396	62.0	54.80	314 (79.3)	40 (11.3)	1048	30.1 (0–90)	891 (85.0)	24.3 (0.081–144)

* Days elapsed since symptom onset.

COI, cut-off index.

The anti-SARS-CoV-2 antibody positivity rate and COI differed significantly among the six institutions. However, after stratifying the results by number of weeks since symptom onset, there were no statistically significant differences among the six institutions (S1–S4 Figs).

### Anti-SARS-CoV-2 antibody level over time since symptom onset

**Fig 1:**
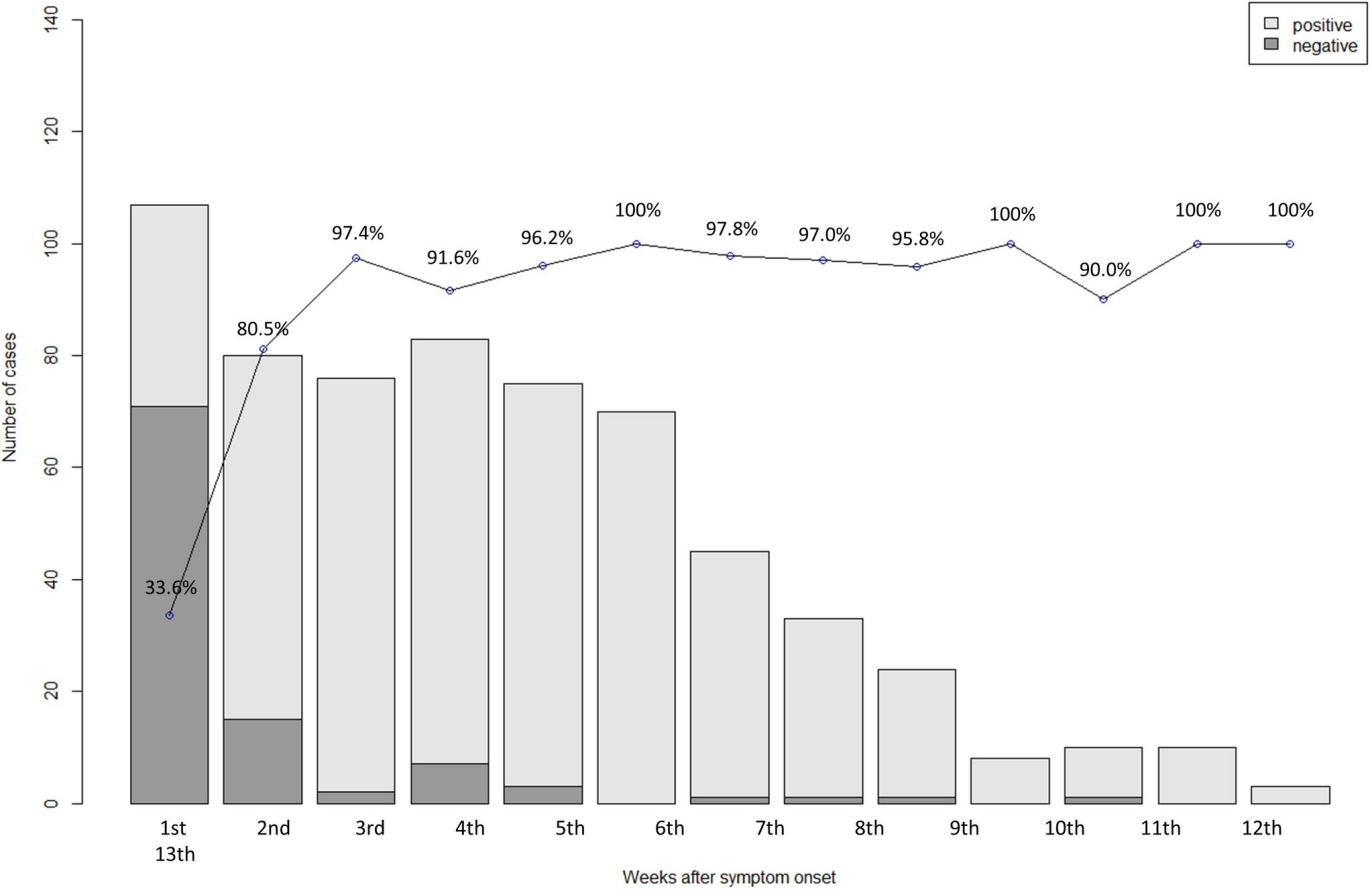
Anti-SARS-CoV-2 antibody positivity rate over time after symptom onset. Numbers above bars are positivity rates.

The anti-SARS-CoV-2 antibody positivity rate increased with time since symptom onset (p < 0·001). In week 1 of symptoms, the positivity rate was 33·6%, 97·4% in week 3, and 100% from weeks 6 to 13 (Fig 1 and S1 Table).

**Fig 2:**
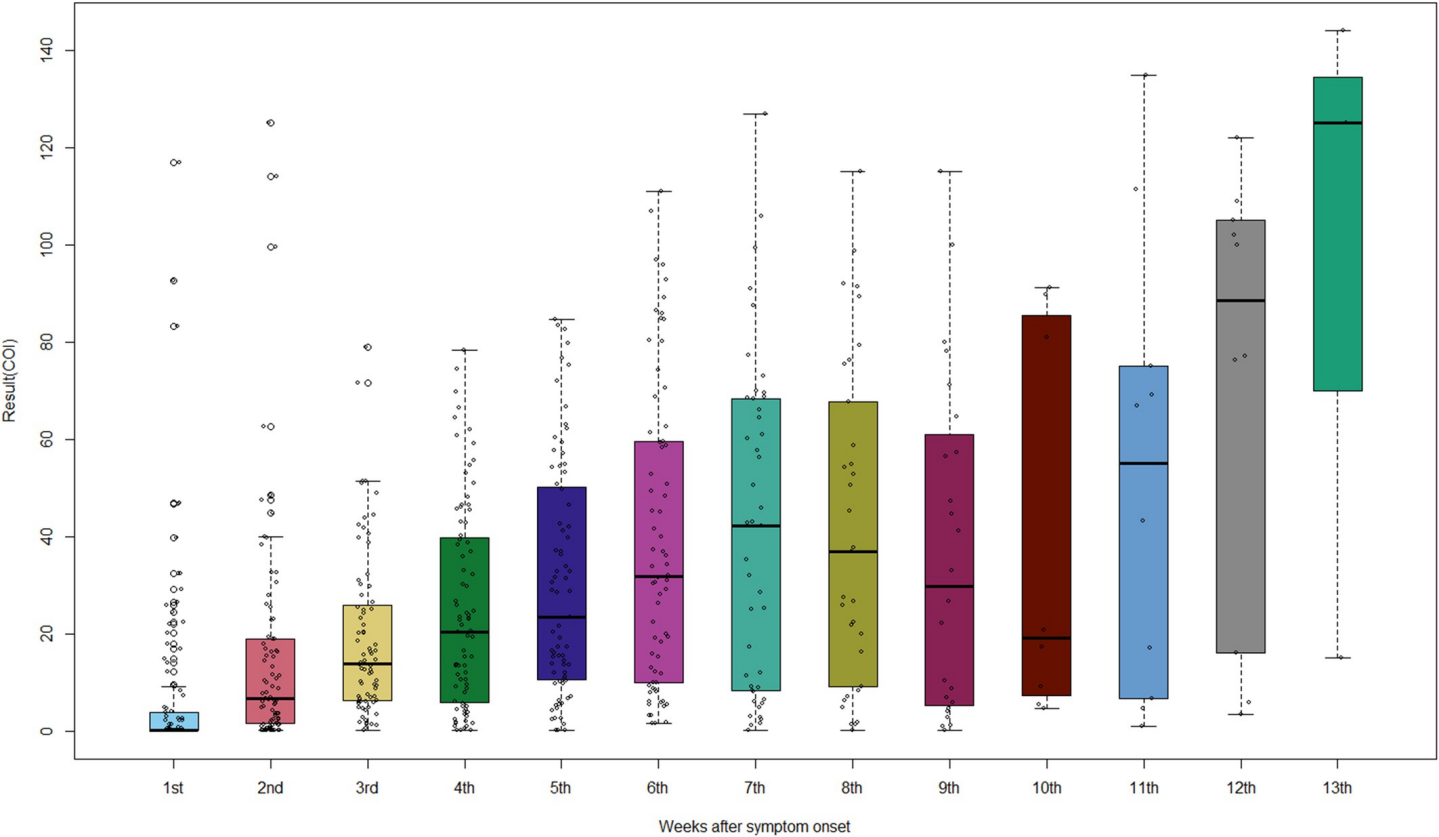
COI of anti-SARS-CoV-2 antibody over time since symptom onset. Data are means with 95% confidence intervals.

The COI increased significantly with time since symptom onset (p < 0·001) and remained high up to week 13 (Fig 2 and S2 Table). A significant correlation between COI and time elapsed since symptom onset was observed (rho = 0·3439, p < 0·001) (S5 Fig).

### Anti-SARS-CoV-2 antibody positivity according to clinical characteristics

**Fig 3:**
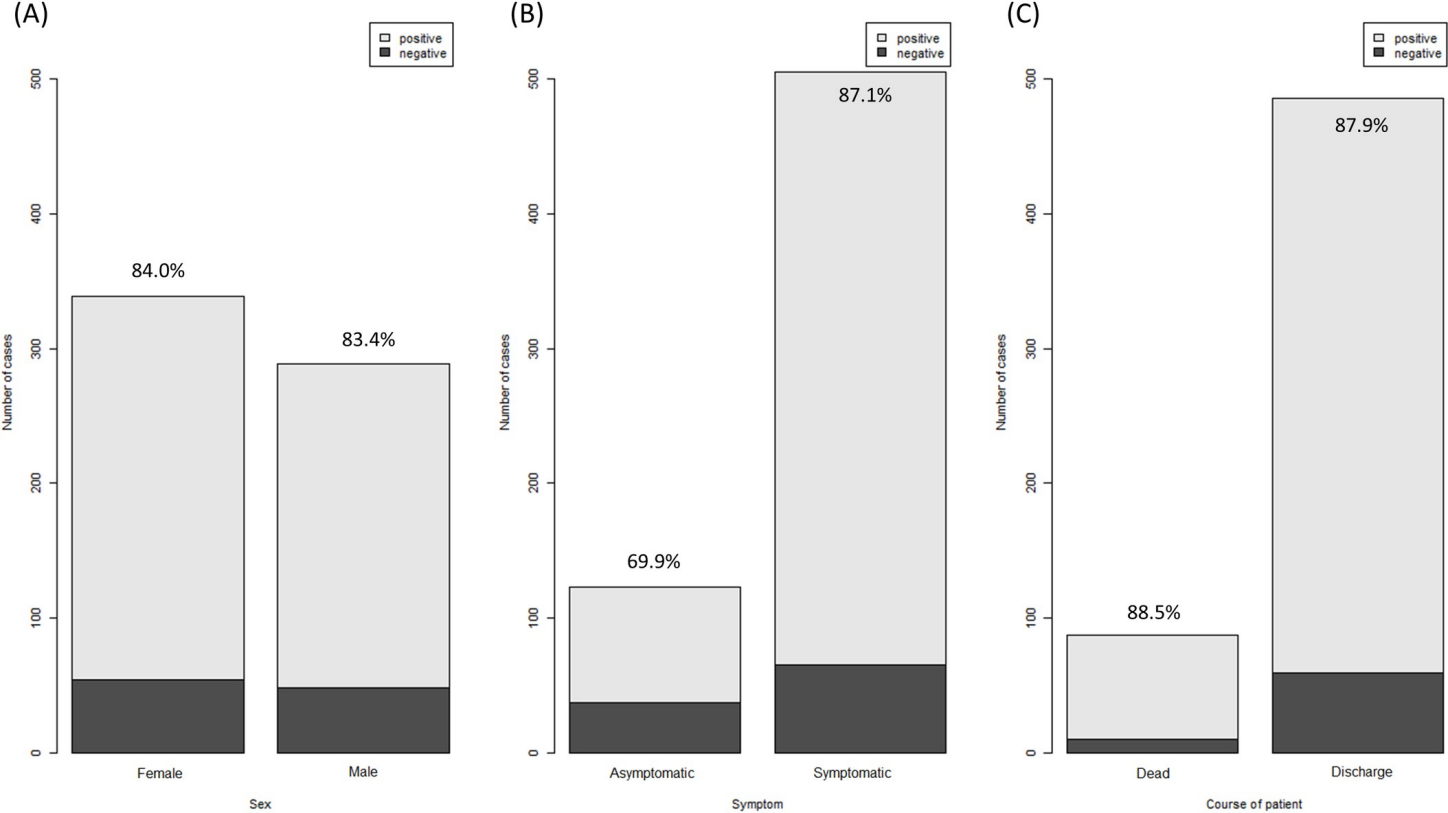
Anti-SARS-CoV-2 antibody positivity rate according to sex (A), symptoms (B), and disease course (C). Numbers above bars are positivity rates.

**Fig 4:**
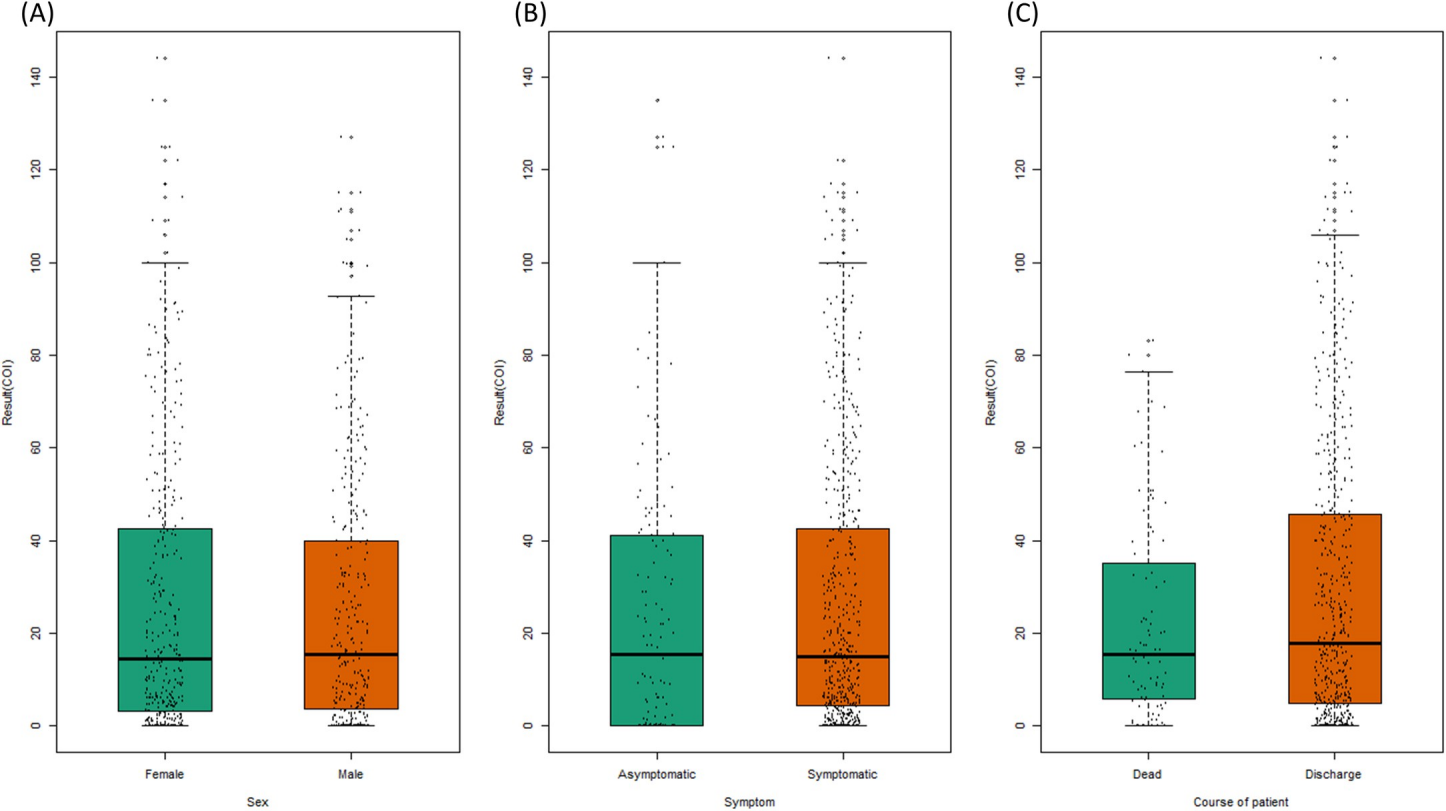
COI of anti-SARS-CoV-2 antibody according to sex (A), presence of symptoms (B) and disease course (C). Data are means with 95% confidence intervals.

There was no significant difference in anti-SARS-CoV-2 antibody positivity rate according to sex and disease course (Fig 3). However, there was a significant difference in positivity rates between patients with and without symptoms (87·1% *vs*. 69·9%, p < 0·001). After stratifying the results by number of weeks elapsed since symptom onset, there was no significant difference in positivity rate by sex, presence of symptoms, and disease course (S6–S8 Figs). There was no significant difference in COI according to sex, symptoms, and disease course (Fig 4).

### Pattern of anti-SARS-CoV-2 antibody level in consecutive samples

A total of 119 patients had samples in at least two different weeks (S9 Fig). Based on the COI from weeks 1 to 5, patients were classified as having an ascending or descending pattern according to the slope of the linear regression equation (slope > 0, ascending; < 0, descending).

**Fig 5:**
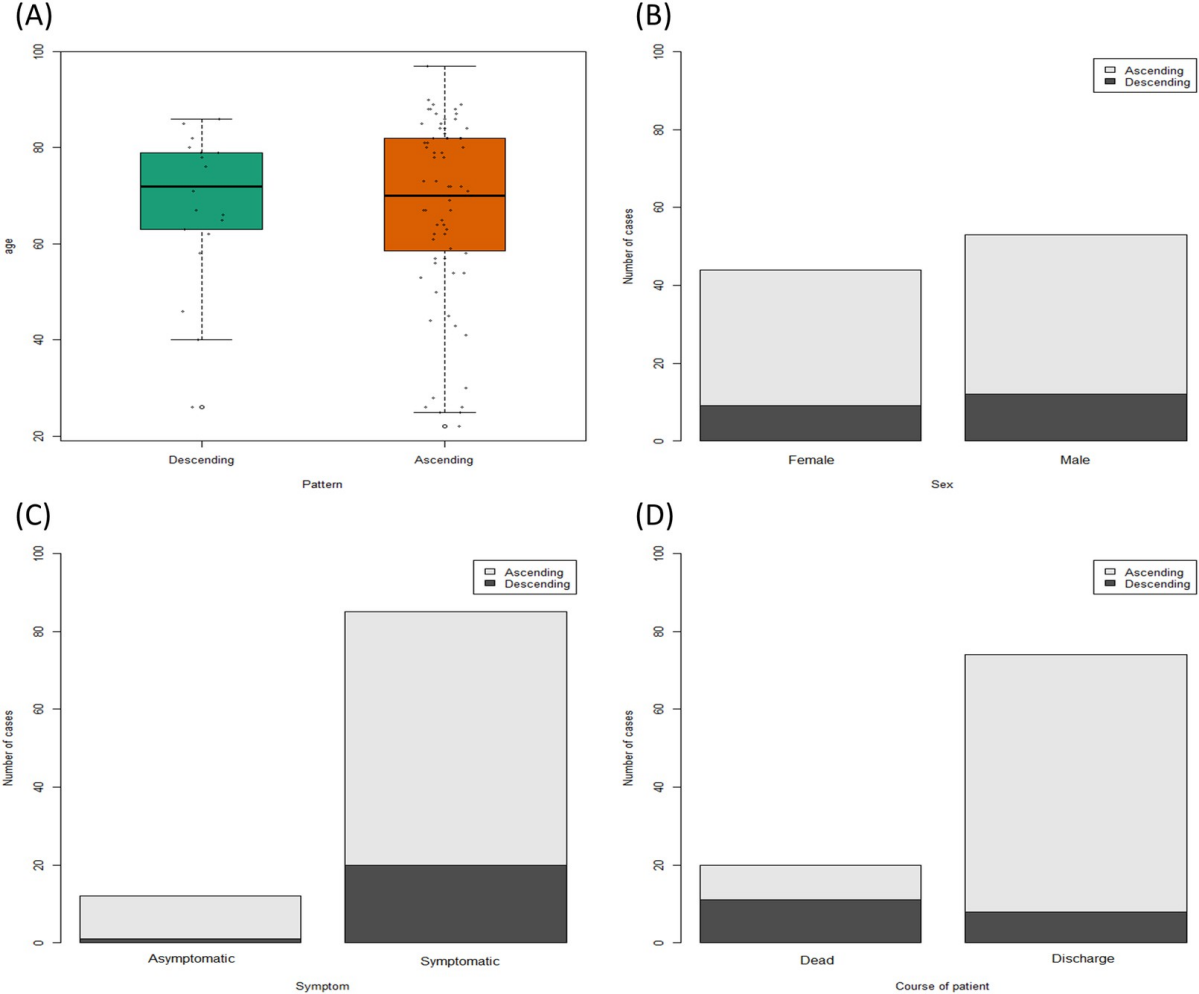
Anti-SARS-CoV-2 antibody pattern across consecutive samples. (A) Ages of patients with the ascending and descending patterns. (B) Ascending pattern according to sex, (C) presence of symptoms, and (D) disease course.

There was no difference in age between patients with the ascending and descending patterns (Fig 5). In addition, there was no difference in the proportion of patients with the ascending pattern according to sex and presence of symptoms. However, there was a significant difference in the proportion of the ascending pattern between deceased and discharged patients (45% *vs*. 89·2%, p < 0·001).

### Comparison of serology and real-time RT-PCR

The *RdRP* Ct value increased with time after symptom onset (rho = 0·563, p < 0·001) (S10 Fig). A significant correlation between the anti-SARS-CoV-2 antibody COI and the *RdRP* Ct value was observed (rho = 0·197, p < 0·001) (S11 Fig).

### Factors affecting anti-SARS-CoV-2 antibody positivity

By logistic regression analysis, the anti-SARS-CoV-2 antibody positivity rate increased with time after symptom onset and as the *RdRP* Ct value increased (Table 2). However, the positivity rate was not related to age, presence of symptoms, and disease course.

**Table 2:** Factors affecting the anti-SARS-CoV-2 antibody positivity rate.

Factors	Odds Ratio	Lower CI (95%)	Upper CI (95%)	p-Value
Weeks	3.25	2.30	4.59	0.0000
Age	0.99	0.97	1.01	0.3938
Sex	1.49	0.70	3.16	0.3030
Symptoms	2.03	0.78	5.29	0.1466
Disease course	0.95	0.36	2.51	0.9253
*RdRP* Ct value	1.10	1.03	1.17	0.0025

CI, confidence interval; Ct, cycle threshold.

## Discussion

Various antibody assays for SARS-CoV-2 have been approved for use, but their performances are variable [4, 10–13]. A recent meta-analysis reported that most antibody tests showed a low sensitivity in the first week since symptom onset (< 30·1%), a tendency to increase in the second week (70%), and positive in almost all confirmed COVID-19 cases beginning in the third week (more than 90%) [14]. The antibody positivity rate in this work was 33·6% in week 1, 77·4% in week 2, and 97% in week 3 after symptom onset in patients with SARS-CoV-2 which was similar to that of prior meta-analysis.

However, few studies estimated the sensitivity of tests beyond 5 weeks after symptom onset. A study involving serial measurements of anti-SARS-CoV-2 IgG, the first and last of which were an average of 37 and 86 days, respectively, after symptom onset, showed a decreased antibody concentration and a half-life of 36 days [15]. By contrast, other studies have reported that the anti-SARS-CoV-2 antibody level remains high for 50 to 60 days after symptom onset and is only slightly decreased at 120 days [16, 17]. As with the latter, our studies also showed 100% of positive rate at week 6 and > 90% at week 13. In addition, the antibody COI was high until week 13, suggesting that anti-SARS-CoV-2 antibody persisted for at least 13 weeks.

The results of studies on the correlation between symptoms and anti-SARS-CoV-2 antibody positivity in patients with COVID-19 differ according to antibody class and target [18–20]. Weisberg *et al*. analyzed serum samples from 19 adults recruited as convalescent plasma donors who recovered from mild COVID-19 in comparison with serum of adults hospitalized with severe COVID-19 [18]. The concentrations of IgG, IgM, and IgA against S protein, but not that of IgG against N protein, were significantly higher in patients with severe COVID-19. William *et al*. reported that the IgG level increased early only in patients with severe COVID-19, but the IgM level increased soon after symptom onset of both mild and severe COVID-19 [19]. A recent study of 37 asymptomatic patients reported that they had a significantly longer viral shedding duration and lower virus-specific IgG level in the acute phase than symptomatic patients [20]. We found a significant difference in the anti-SARS-CoV-2 antibody positivity rate between symptomatic and asymptomatic patients. However, after stratification by number of weeks elapsed since symptom onset, there was no significant difference in anti-SARS-CoV-2 antibody positivity according to the presence and absence of symptoms. In addition, there were more samples from symptomatic patients with a long time elapsed since symptom onset compared to asymptomatic patients (S12 Fig). This is in agreement with a prior report that the virologic remission period of symptomatic patients is significantly longer than that of asymptomatic patients [21].

In the recent studies about association between prognosis of COVID-19 and anti-SARS-CoV-2 antibody titer, non-structural protein targeted IgM antibodies were associated with a good prognosis and structural protein, including N protein, targeted IgG antibodies were associated with high mortality [22]. Although the kit we used measured structural protein targeted total antibodies including IgG, there was no significant difference in anti-SARS-CoV-2 antibody between patients who died and those who recovered from COVID-19. However, analysis of serial samples showed that a descending COI pattern was significantly more frequent among patients who died than among those who recovered. Therefore, a decreasing anti-SARS-CoV-2 antibody COI pattern in the early stage of COVID-19 may be associated with a poor prognosis. This finding would be helpful in clinical and public health settings in that it is possible to predict the patient’s prognosis and implement appropriate management through continuous simple antibody testing.

In a logistic regression analysis, the antibody positivity rate increased with increasing *RdRP* Ct value. However, the *RdRP* Ct value increased with time elapsed after symptom onset (S13 Fig), suggesting an effect of time. Therefore, there was no correlation between SARS-CoV-2 antibody and viral load.

This study had several limitations. We used one anti-SARS-CoV-2 antibody assay targeting the nucleocapsid protein of SARS-CoV-2. The small number of blood samples for each week after symptom onset hampers generalization of the results. The COI was not designed for quantitative analysis, so the COI results cannot be generalized. In this study, the antibody titer was compared with whether the patient died or was recovered and discharged, but the severity of symptoms was not considered. Further researches on these would be needed. Although having these limitation, this study conducted a multiple comparative tests collecting specimens from 6 different institutions to evaluate the serological status of COVID-19 patients in South Korea. In conclusion, anti-SARS-CoV-2 antibody persisted, and the level increased for 13 weeks after symptom onset with high COI. Also, a decreasing antibody COI pattern in the early stages of infection (up to 5 weeks) might be related to a poor prognosis of COVID-19. As new treatments and vaccines are introduced, it is important to monitor continuously the usefulness of anti-SARS-CoV-2 antibody assays.

## Supporting information

S1 Fig Anti-SARS-CoV-2 antibody positivity rates of the institutions. (TIF)

S2 Fig COI values of anti-SARS-CoV-2 antibody. (TIF)

S3 Fig Anti-SARS-CoV-2 antibody positivity rate stratified by time since symptom onset. (TIF)

S4 Fig COI values of anti-SARS-CoV-2 antibody stratified by time since symptom onset. (TIF)

S5 Fig Correlation between the COI value of anti-SARS-CoV-2 antibody and number of days since symptom onset. The red line represents the linear regression equation (slope = 0.5131, adjusted R^2^ = 0.1184).
(TIF)

S6 Fig Anti-SARS-CoV-2 antibody positivity rate according to sex stratified by time since symptom onset. (TIF)

S7 Fig Anti-SARS-CoV-2 antibody positivity rate according to presence of symptoms stratified time since symptom onset. (TIF)

S8 Fig Anti-SARS-CoV-2 antibody positivity rate according to disease course stratified time since symptom onset. (TIF)

S9 Fig COI values of anti-SARS-CoV-2 antibody in consecutive samples from 119 patients. (TIF)

S10 Fig Correlation between the cycle threshold of *RdRP* and number of days since symptom onset. The red line represents the linear regression equation (slope = 0.2239, adjusted R^2^ = 0.2218).
(TIF)

S11 Fig Correlation between the COI value of anti-SARS-CoV-2 antibody and the cycle threshold value of *RdRP*. The red line represents the linear regression equation (slope = 0.6356, adjusted R^2^ = 0.01784).
(TIF)

S12 Fig Days from the symptom onset of latest sample according to symptom. (TIF)

S13 Fig Correlation between the days from the symptom onset of samples and the cycle threshold value of *RdRP*. The red line represents the linear regression equation (slope = 0.1218, adjusted R^2^ = 0.0885).
(TIF)

S1 Table Anti-SARS-CoV-2 antibody positivity rate according to time since symptom onset. (DOCX)

S2 Table Post hoc analysis of the COI value of anti-SARS-CoV-2 antibody and time since symptom onset. (DOCX)
