# Supplementary figures and images for: Serological evaluation of patients with coronavirus disease-2019 in Daegu, South Korea
Source: PLoS One. 2022 Jan 20;17(1):e0262820. doi: 10.1371/journal.pone.0262820 (PMC8775192; doi:10.1371/journal.pone.0262820)

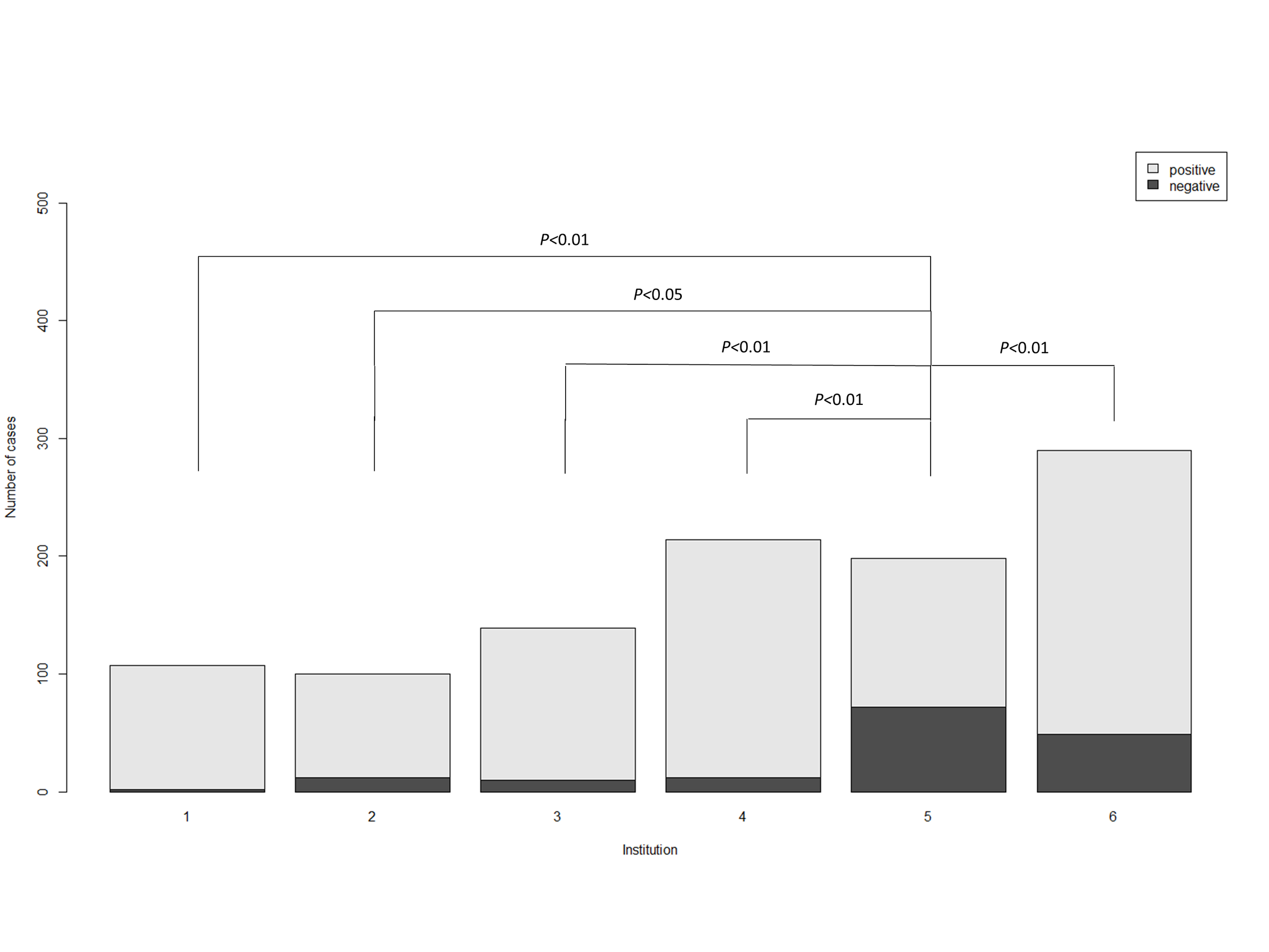

Supplement: S1 Fig — (TIF) [file pone.0262820.s001.tif]

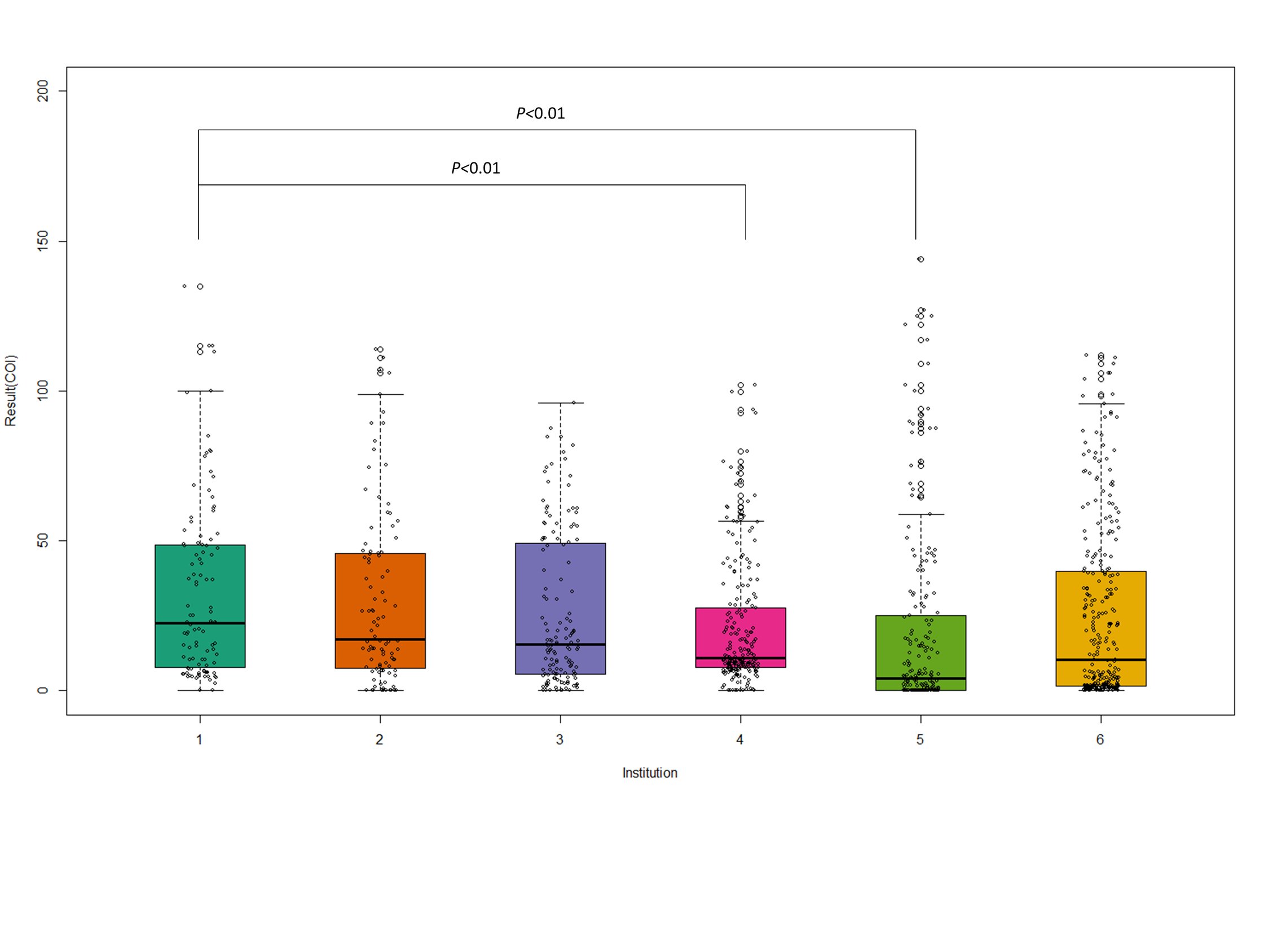

Supplement: S2 Fig — (TIF) [file pone.0262820.s002.tif]

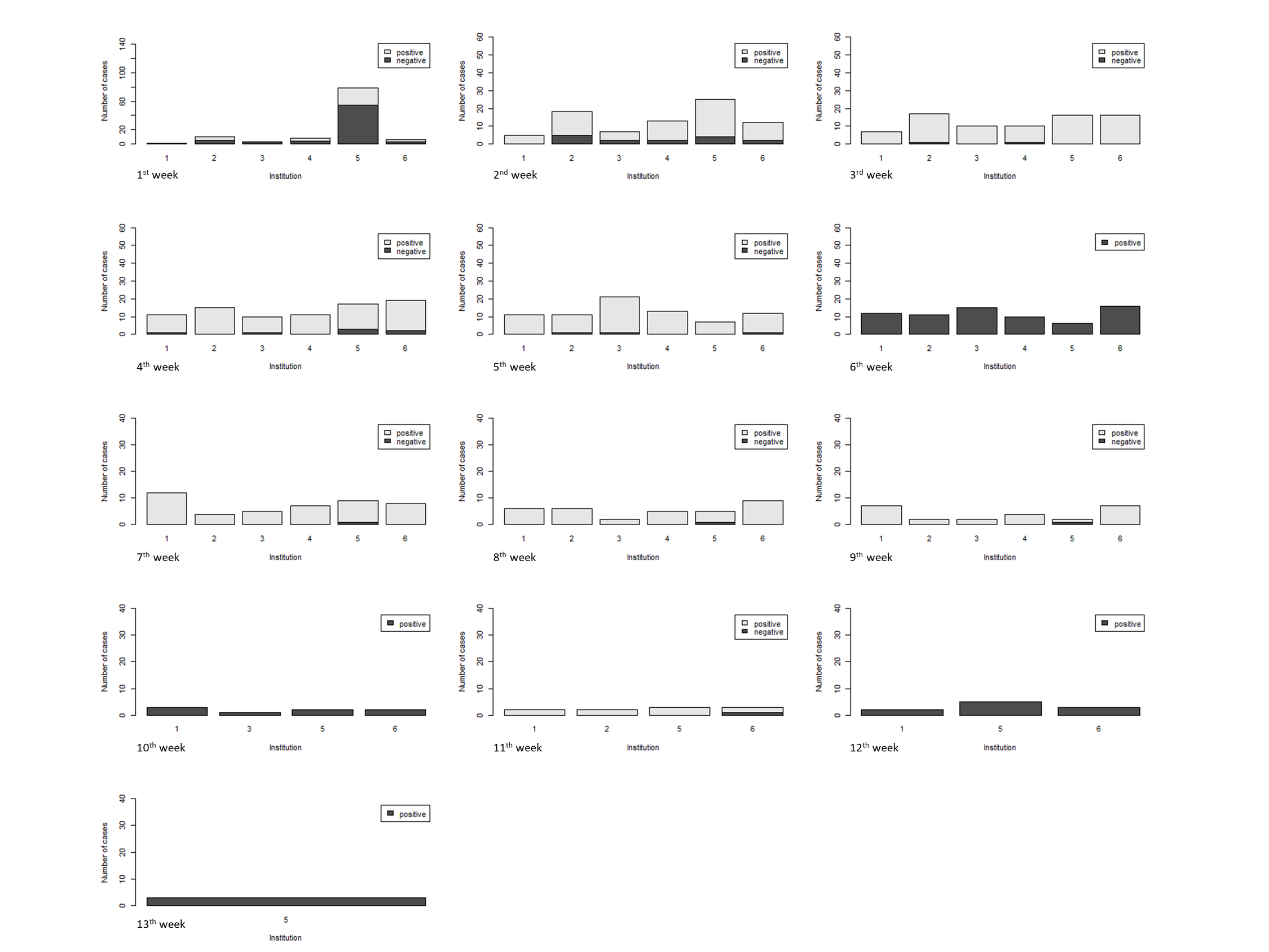

Supplement: S3 Fig — (TIF) [file pone.0262820.s003.tif]

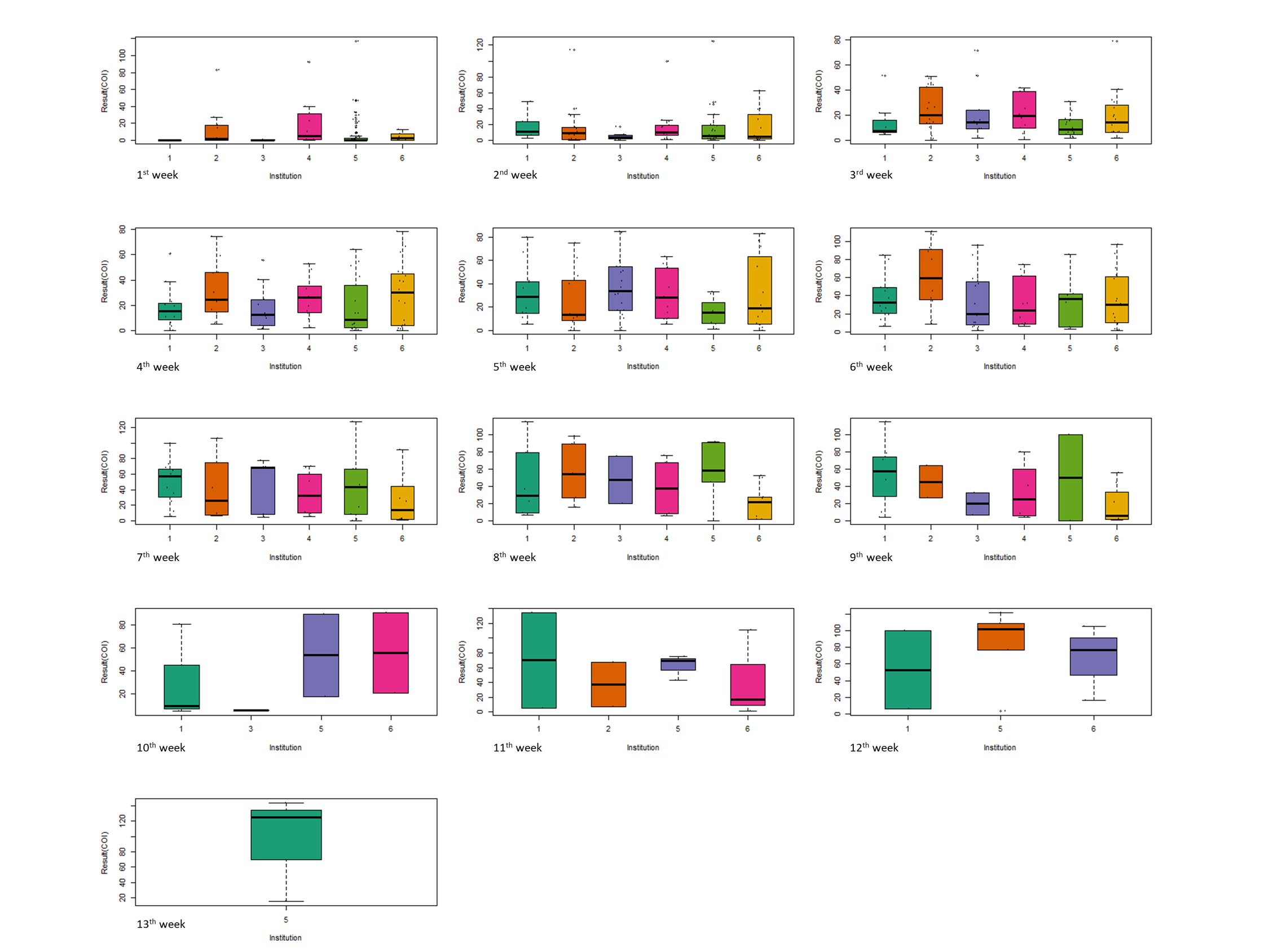

Supplement: S4 Fig — (TIF) [file pone.0262820.s004.tif]

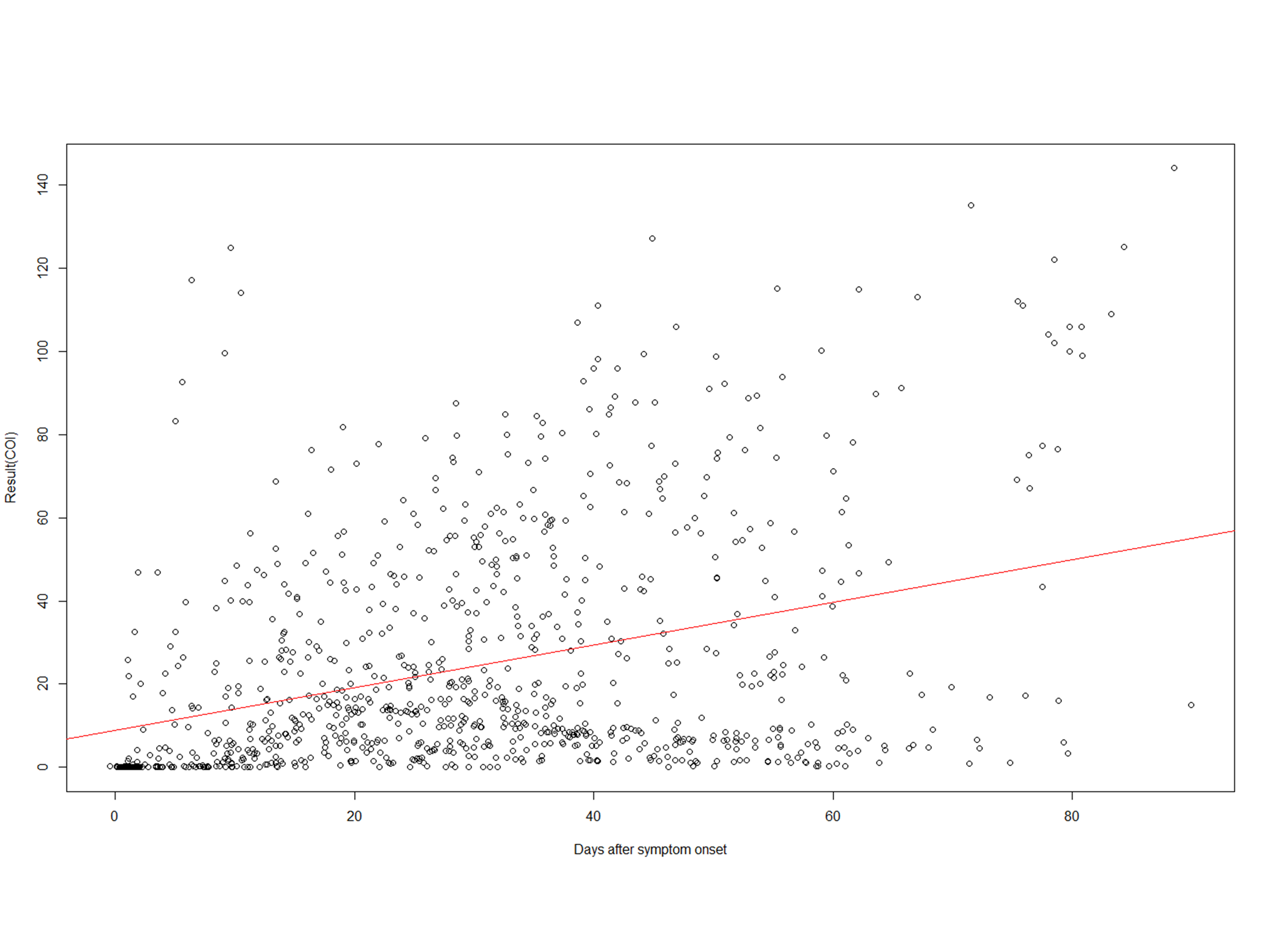

Supplement: S5 Fig — The red line represents the linear regression equation (slope = 0.5131, adjusted R2 = 0.1184). (TIF) [file pone.0262820.s005.tif]

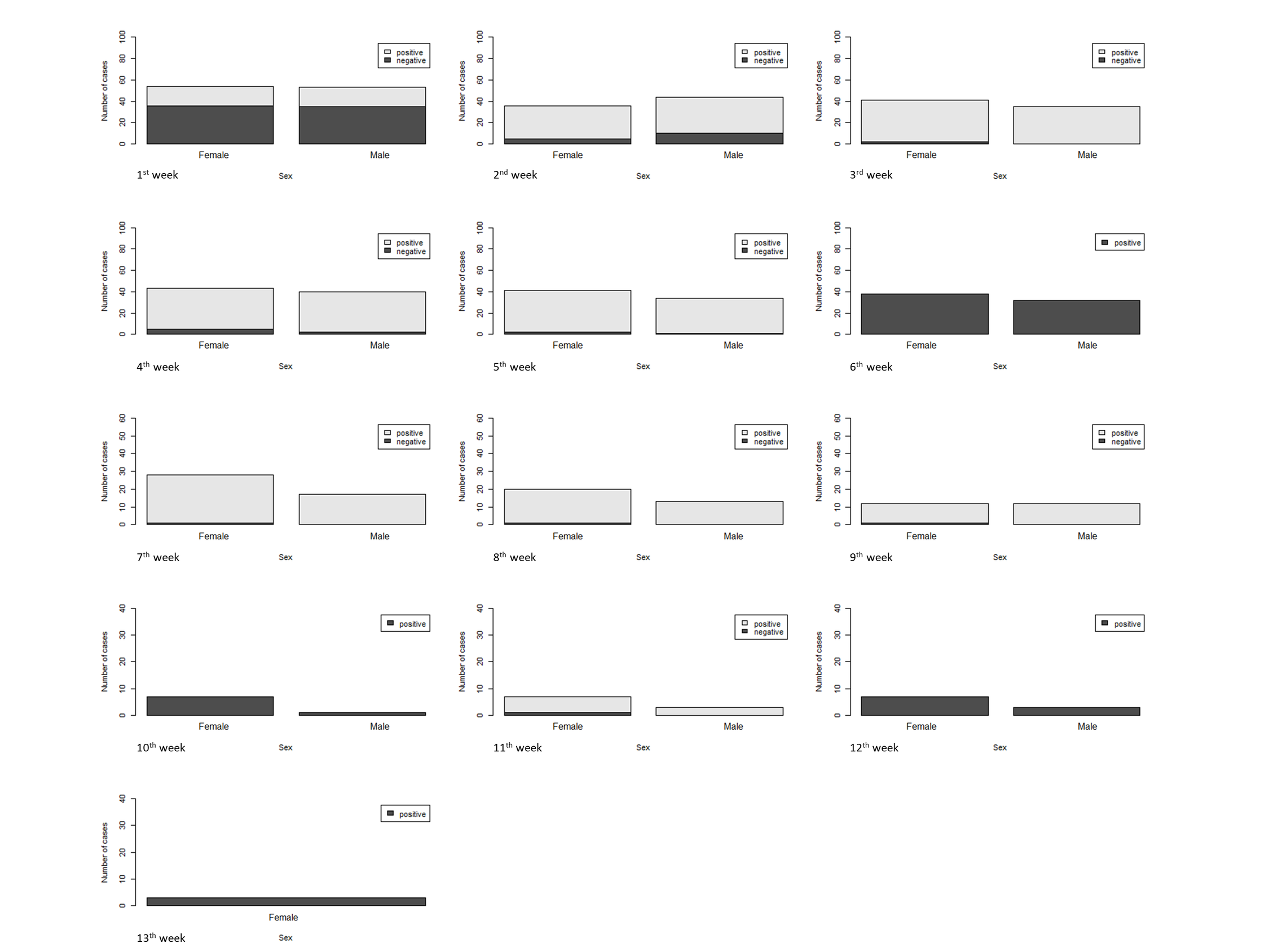

Supplement: S6 Fig — (TIF) [file pone.0262820.s006.tif]

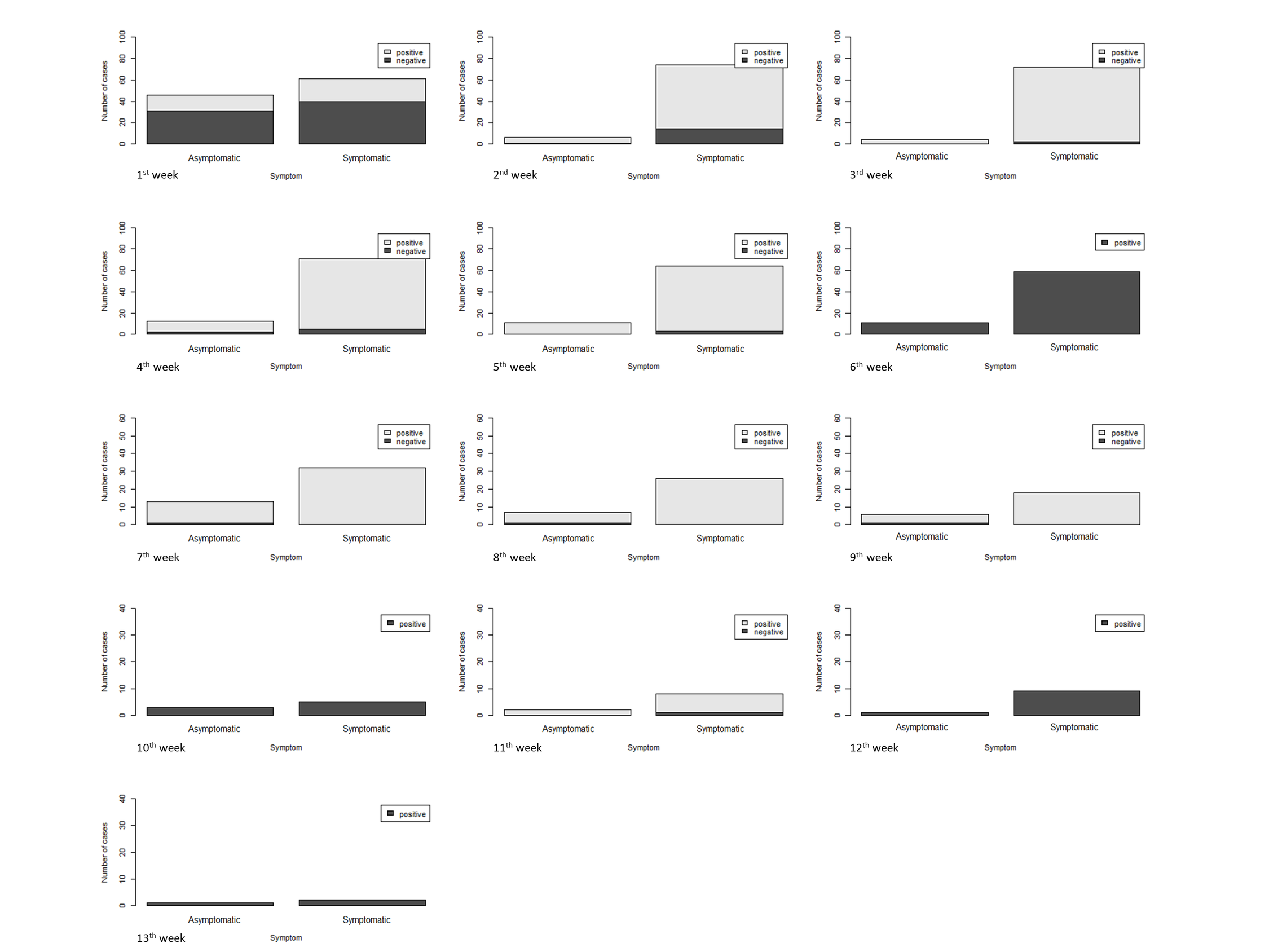

Supplement: S7 Fig — (TIF) [file pone.0262820.s007.tif]

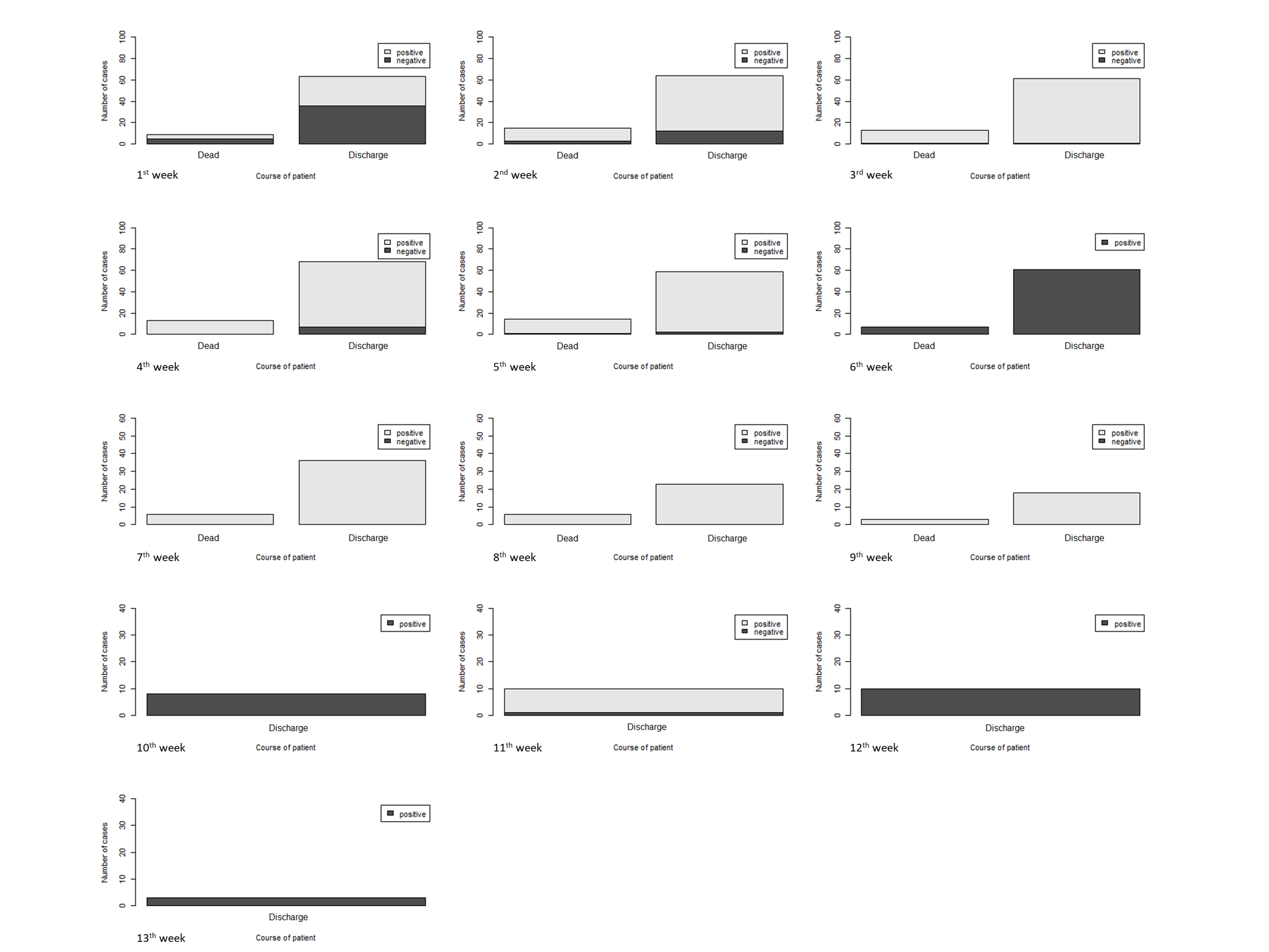

Supplement: S8 Fig — (TIF) [file pone.0262820.s008.tif]

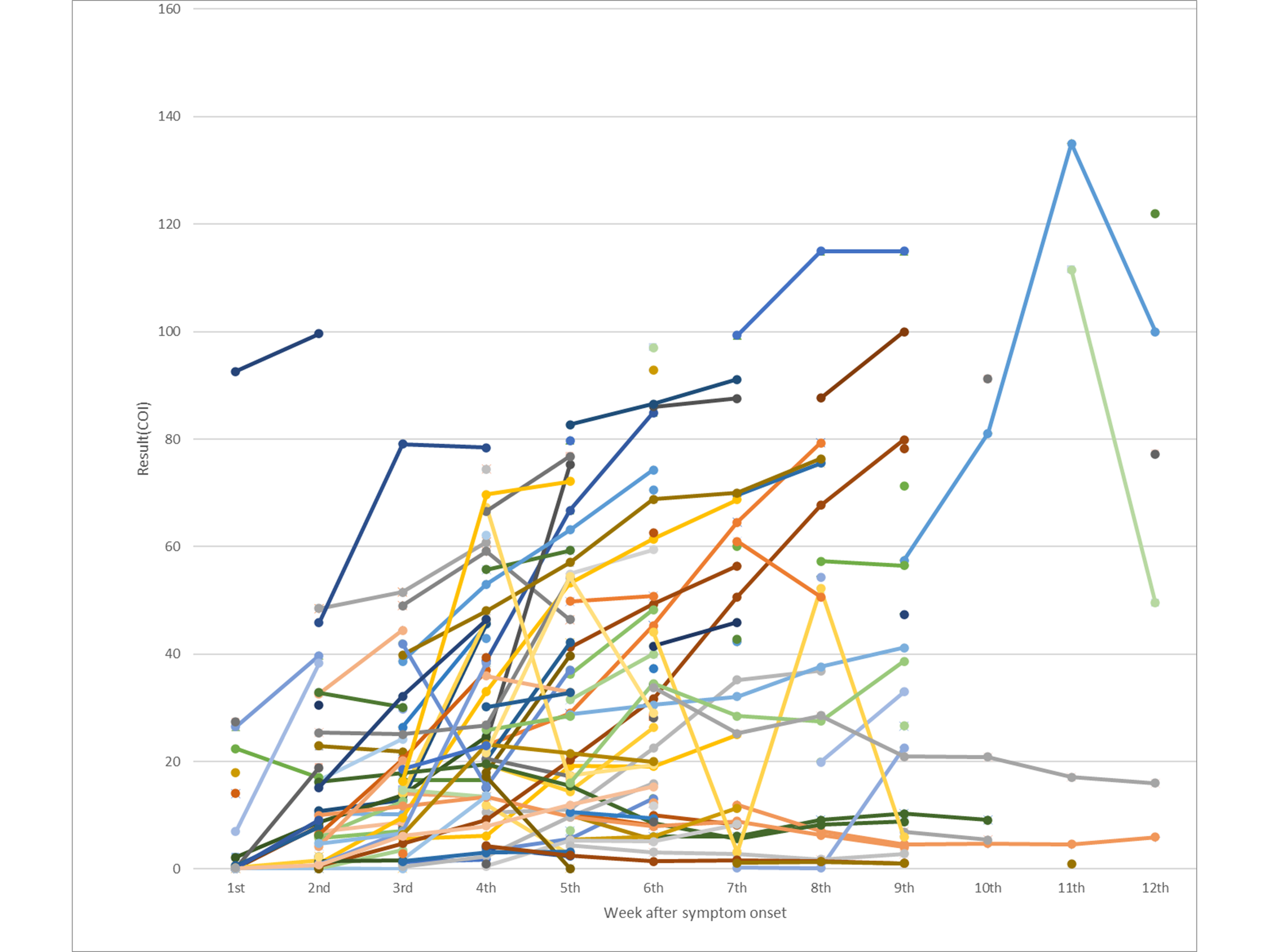

Supplement: S9 Fig — (TIF) [file pone.0262820.s009.tif]

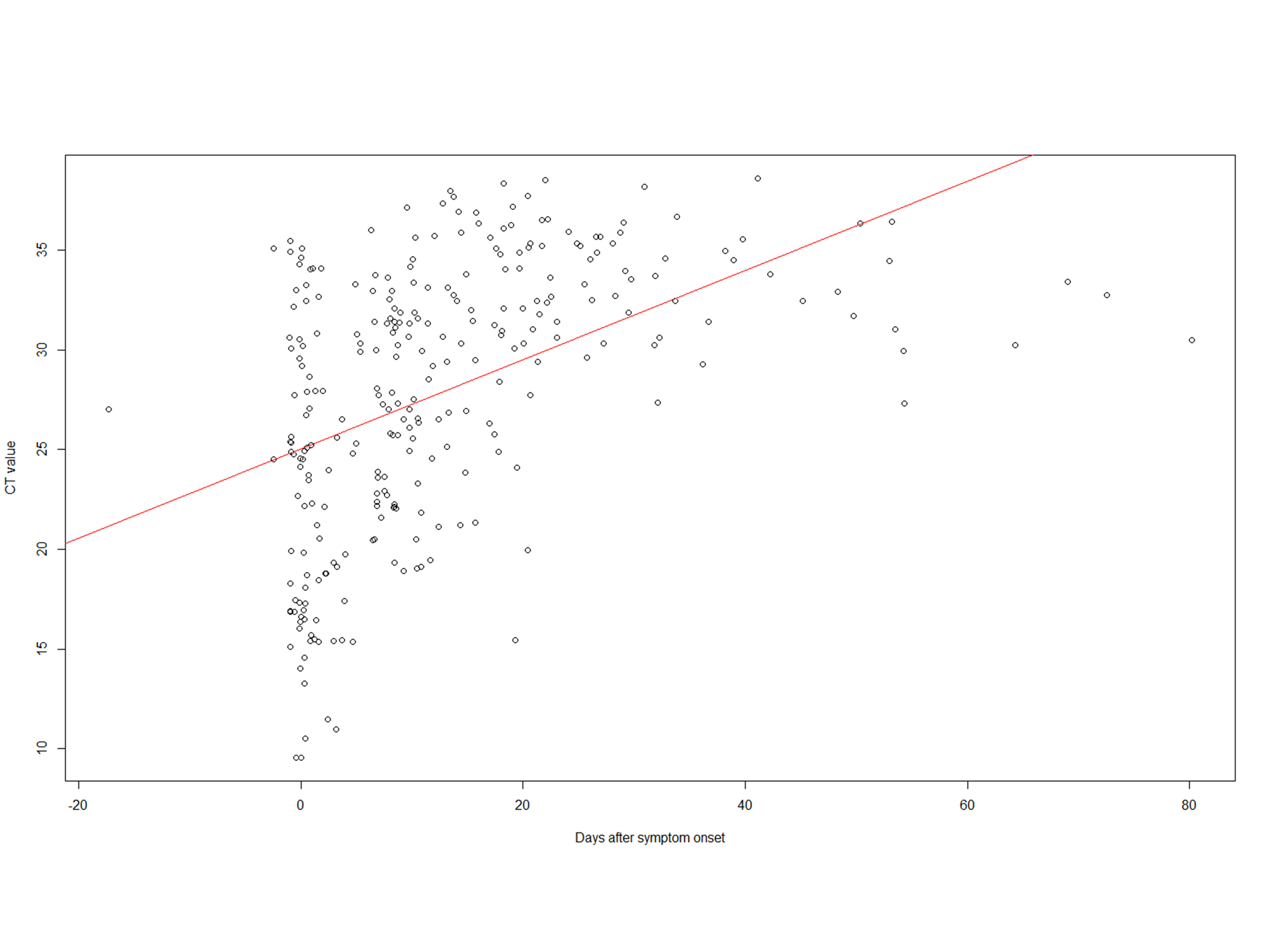

Supplement: S10 Fig — The red line represents the linear regression equation (slope = 0.2239, adjusted R2 = 0.2218). (TIF) [file pone.0262820.s010.tif]

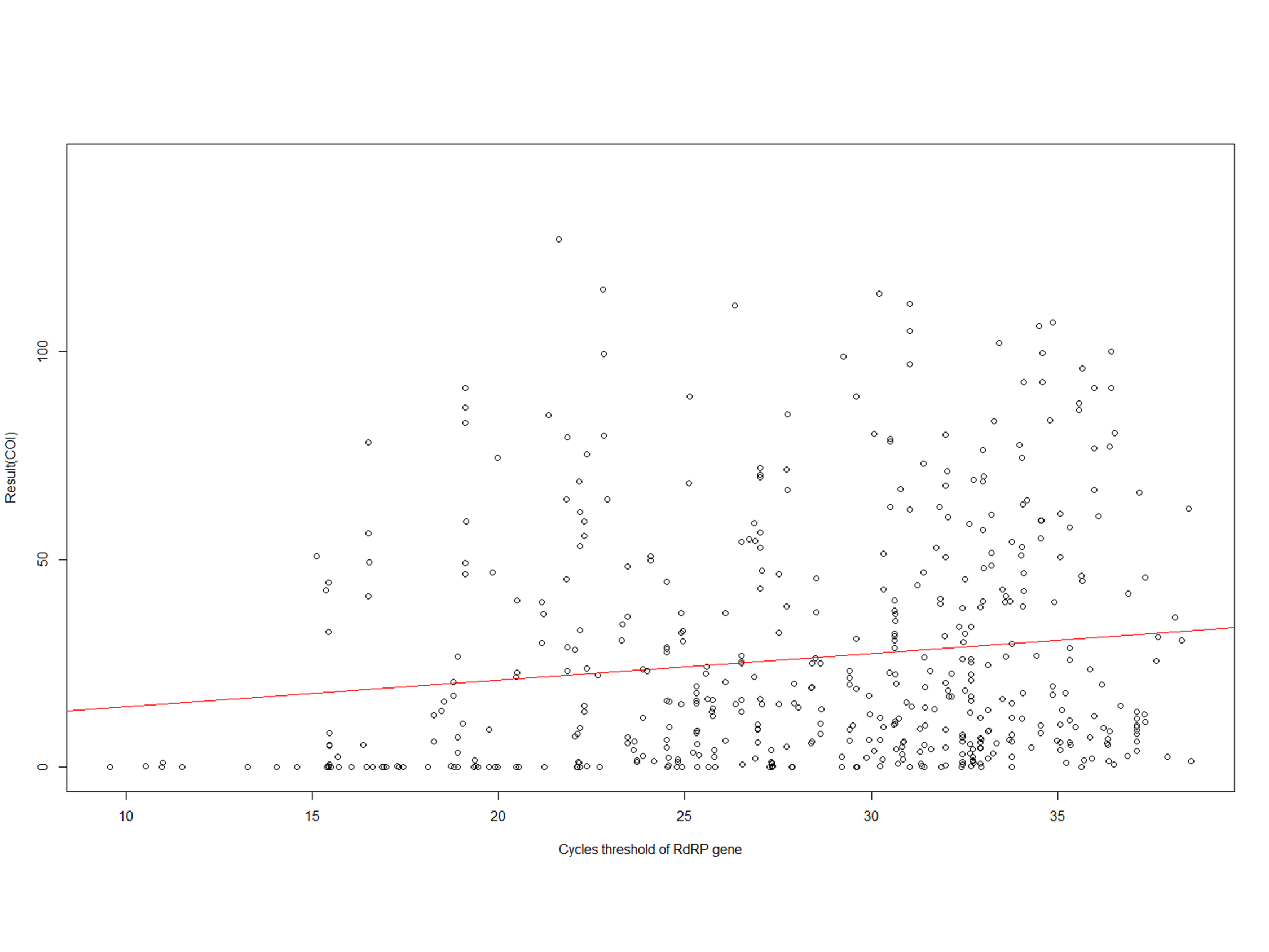

Supplement: S11 Fig — The red line represents the linear regression equation (slope = 0.6356, adjusted R2 = 0.01784). (TIF) [file pone.0262820.s011.tif]

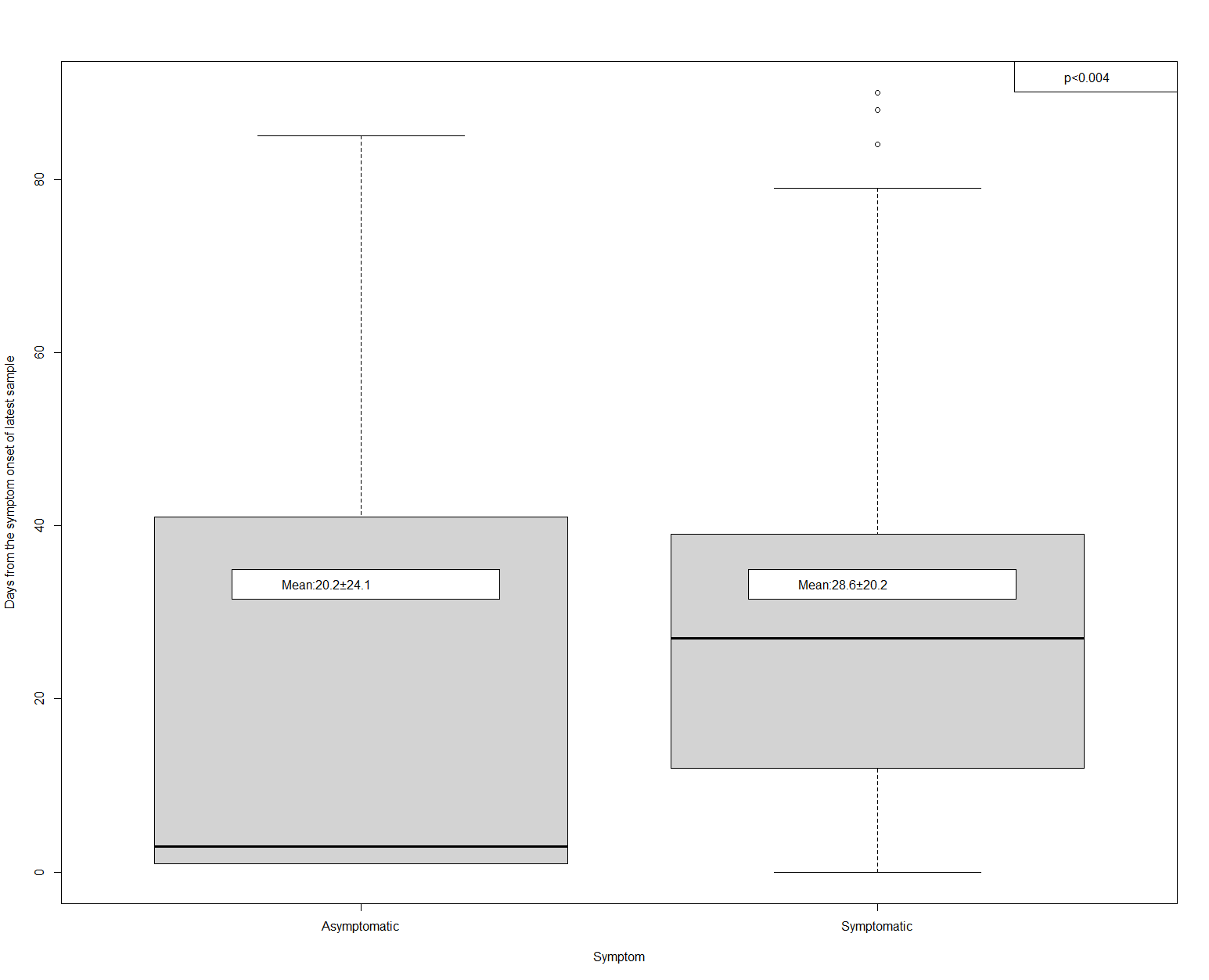

Supplement: S12 Fig — (TIF) [file pone.0262820.s012.tif]

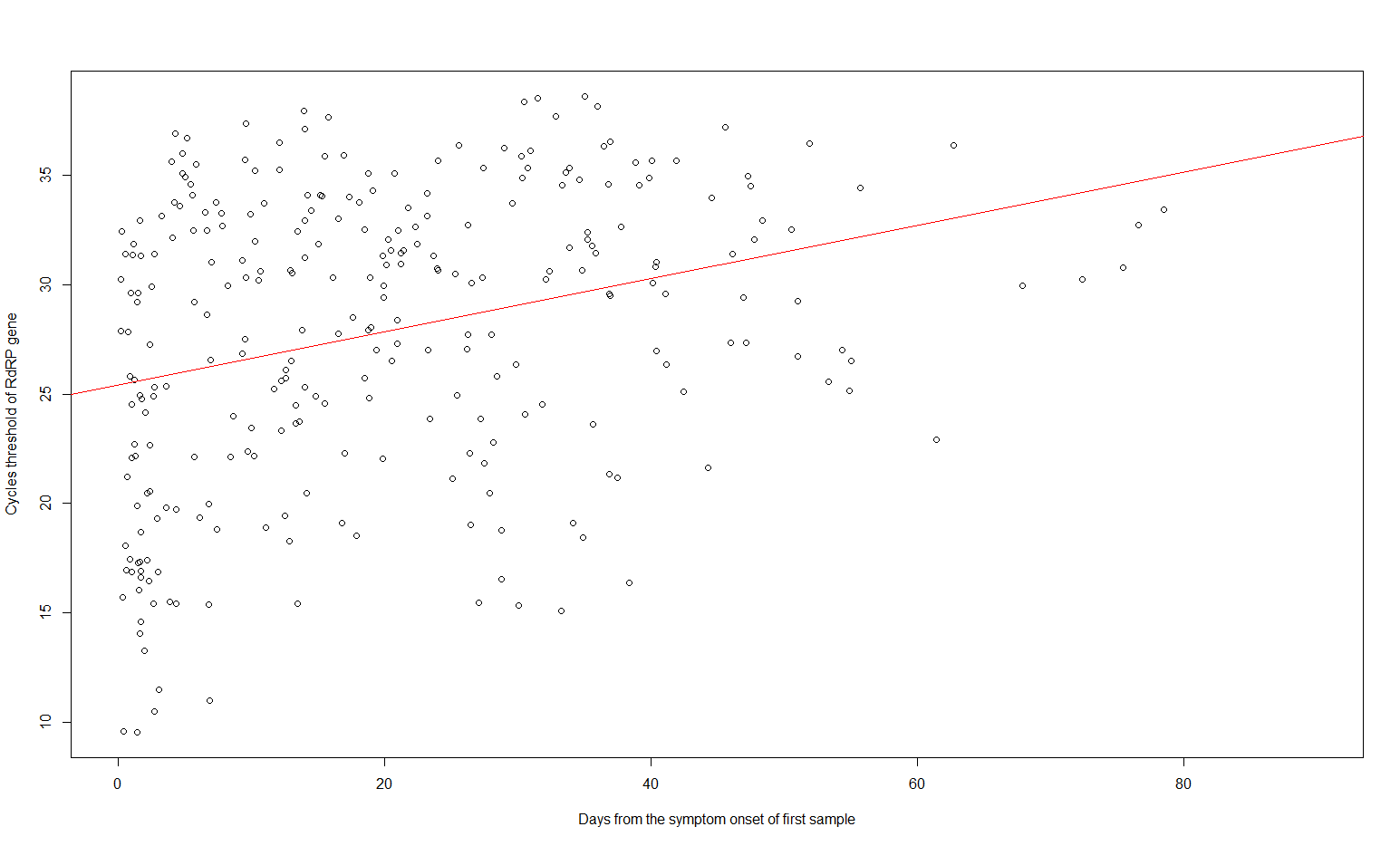

Supplement: S13 Fig — The red line represents the linear regression equation (slope = 0.1218, adjusted R2 = 0.0885). (TIF) [file pone.0262820.s013.tif]
