# Supplementary material for: Serological evaluation of patients with coronavirus disease-2019 in Daegu, South Korea
Source: PLoS One. 2022 Jan 20;17(1):e0262820. doi: 10.1371/journal.pone.0262820 (PMC8775192; doi:10.1371/journal.pone.0262820)
Supplement: S1 Table — (DOCX) [file pone.0262820.s014.docx]

**S1 Table.** Anti-SARS-CoV-2 antibody positivity rate according to time since symptom onset.

| **Weeks** | **1st** | **2nd** | **3rd** | **4th** | **5th** | **6th** | **7th** | **8th** | **9th** | **10th** | **11st** | **12th** | **13th** |
| --- | --- | --- | --- | --- | --- | --- | --- | --- | --- | --- | --- | --- | --- |
| **Number of**  **negative results** | 71 | 15 | 2 | 7 | 3 | 0 | 1 | 1 | 1 | 0 | 1 | 0 | 0 |
| **Number of**  **positive results** | 36 | 65 | 74 | 76 | 72 | 70 | 44 | 32 | 23 | 8 | 9 | 10 | 3 |
| **Total** | 107 | 80 | 76 | 83 | 75 | 70 | 45 | 33 | 24 | 8 | 10 | 10 | 3 |
| **Positive rate (%)** | 33.6 | 81.3 | 97.4 | 91.6 | 96.0 | 100.0 | 97.8 | 97.0 | 95.8 | 100.0 | 90.0 | 100.0 | 100.0 |
