# Supplementary material for: Serological evaluation of patients with coronavirus disease-2019 in Daegu, South Korea
Source: PLoS One. 2022 Jan 20;17(1):e0262820. doi: 10.1371/journal.pone.0262820 (PMC8775192; doi:10.1371/journal.pone.0262820)
Supplement: S2 Table — (DOCX) [file pone.0262820.s015.docx]

**S2 Table.** Post hoc analysis of the COI value of anti-SARS-CoV-2 antibody and time since symptom onset.

|  | **Difference** | **Lower CI (95%)** | **Upper CI (95%)** | **p-value** |
| --- | --- | --- | --- | --- |
| **3 vs 1** | 11.341 | 2.4494 | 20.2 | 0.002 |
| **4 vs 1** | 17.032 | 7.1584 | 26.9 | <0.001 |
| **5 vs 1** | 22.95 | 11.6526 | 34.2 | <0.001 |
| **6 vs 1** | 31.442 | 17.5018 | 45.4 | <0.001 |
| **7 vs 1** | 33.538 | 15.0678 | 52 | <0.001 |
| **8 vs 1** | 34.119 | 12.4696 | 55.8 | <0.001 |
| **9 vs 1** | 29.203 | 2.7577 | 55.6 | 0.021 |
| **12 vs 1** | 64.061 | 1.6753 | 126.4 | 0.043 |
| **5 vs 2** | 14.7 | 1.6656 | 27.7 | 0.013 |
| **6 vs 2** | 23.191 | 7.8437 | 38.5 | <0.001 |
| **7 vs 2** | 25.288 | 5.7923 | 44.8 | 0.002 |
| **8 vs 2** | 25.868 | 3.3872 | 48.3 | 0.012 |
| **5 vs 3** | 11.61 | 0.0605 | 23.2 | 0.047 |
| **6 vs 3** | 20.101 | 5.9636 | 34.2 | <0.001 |
| **7 vs 3** | 22.197 | 3.5873 | 40.8 | 0.007 |
| **8 vs 3** | 22.778 | 1.0168 | 44.5 | 0.033 |
